# Marine Fungus *Aspergillus chevalieri* TM2-S6 Extract Protects Skin Fibroblasts from Oxidative Stress

**DOI:** 10.3390/md18090460

**Published:** 2020-09-08

**Authors:** Sophia Letsiou, Artemis Bakea, Géraldine Le Goff, Philippe Lopes, Konstantinos Gardikis, Michal Weis, Yehuda Benayahu, Jamal Ouazzani

**Affiliations:** 1 Laboratory of Biochemistry, Research and Development Department, APIVITA S.A., Industrial Park of Markopoulo Mesogaias, Markopoulo Attiki, 19003 Athens, Greece; abakea@hotmail.com (A.B.); gardikis-k@apivita.com (K.G.); 2 Institut de Chimie des Substances Naturelles ICSN, Centre National de la Recherche Scientifique CNRS, Avenue de la Terrasse, 91198 Gif-sur-Yvette, France; geraldine.legoff@cnrs.fr (G.L.G.); Philippe.lopes@cnrs.fr (P.L.); 3 School of Zoology, George S. Wise Faculty of Life Sciences, Tel Aviv University, Ramat Aviv, Tel Aviv 69978, Israel; mich9@tauex.tau.ac.il (M.W.); yehudab@tauex.tau.ac.il (Y.B.)

**Keywords:** *Aspergillus chevalieri*, marine fungi, marine cosmetic ingredient, fibroblast protection, oxidative stress

## Abstract

The strain *Aspergillus chevalieri* TM2-S6 was isolated from the sponge *Axinella* and identified according to internal transcribed spacer (ITS) molecular sequence homology with *Aspergillus* species from the section *Restricti*. The strain was cultivated 9 days on potato dextrose broth (PDB), and the medium evaluated as antioxidant on primary normal human dermal fibroblasts (NHDF). The cultivation broth was submitted to sterile filtration, lyophilized and used without any further processing to give the *Aspergillus chevalieri* TM2-S6 cultivation broth ingredient named ACBB. ACCB contains two main compounds: tetrahydroauroglaucin and flavoglaucin. Under oxidative stress, ACCB showed a significant promotion of cell viability. To elucidate the mechanism of action, the impact on a panel of hundreds of genes involved in fibroblast physiology was evaluated. Thus, ACCB stimulates cell proliferation (VEGFA, TGFB3), antioxidant response (GPX1, SOD1, NRF2), and extracellular matrix organization (COL1A1, COL3A1, CD44, MMP14). ACCD also reduced aging (SIRT1, SIRT2, FOXO3). These findings indicate that *Aspergillus chevalieri* TM2-S6 cultivation broth exhibits significant in vitro skin protection of human fibroblasts under oxidative stress, making it a potential cosmetic ingredient.

## 1. Introduction

Oxidative stress plays a major role in premature skin aging through the production of highly reactive oxygen species (ROS) [1,2,3]. ROS can damage DNA, lipids, proteins and modify transcriptional regulation of genes in skin fibroblasts [3]. Specifically, ROS-induced apoptosis of fibroblasts impacts the production of collagen, elastin and hyaluronic acid [4], resulting in wrinkle formation and skin sagging [5]. This explains the efforts of the academic and industrial sectors to discover bio-based ingredients that protect fibroblasts from ROS-induced damage.

Marine ecosystems represent an untapped reservoir of bio-resources, producing secondary bioactive metabolites and attracting growing interest within the scientific community. Besides marine invertebrates, symbiotic fungi are the main producers of bioactive compounds, some of which have been mistakenly assigned to their hosts [6,7].

*Aspergillus* species are widely used for the production of therapeutic agents [8]. They also produce compounds exhibiting skin related activities like whitening [9], anti-oxidant [10], anti-microbial [11] and UVA screens [12]. Moreover, our previous research indicated that another *Aspergillus* species, *A. puulaauensis*, showed significant in vitro protection of human fibroblasts injured by oxidative stress [13].

*Aspergillus chevalieri* was identified in 1926 and its metabolites reported for potent antioxidant activity [14]. As part of our efforts to develop cosmetics of marine origin, we report in this paper the biological activity of an ingredient issued from the marine fungi *Aspergillus chevalieri* TM2-S6. This strain was isolated in the frame of the EU-funded project TASCMAR, as a symbiont of a mesophotic sponge. To validate the incorporation of this ingredient in cosmetic compositions, we evaluated its protective effect on the premature aging of human fibroblasts by measuring the promotion of cell viability and the impact on genes involved in cellular protection and physiology.

## 2. Results

### 2.1. Phylogeny and Structural Investigation

**Figure 1:**
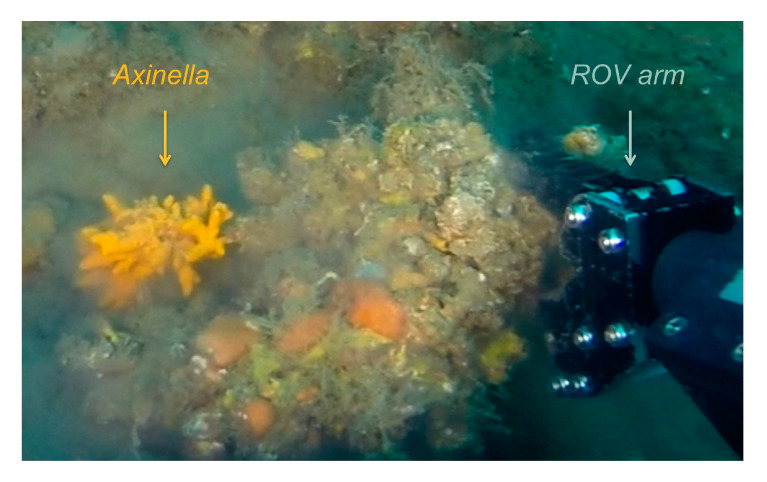
The sponge *Axinella* was collected on the upper mesophotic zone off Tel Aviv–Jaffa, Israeli Mediterranean coast by a ROV arm.

*Aspergillus chevalieri* TM2-S6 was isolated from the sponge *Axinella* collected on the upper mesophotic zone off Tel Aviv–Jaffa, Israeli Mediterranean coast (32°1′42.431″ N 34°46′42.323″ E). The sponge was collected at 38 m depth by a ROV (Remote Operating Vehicle) equipped with a collection arm (Figure 1).

**Figure 2:**
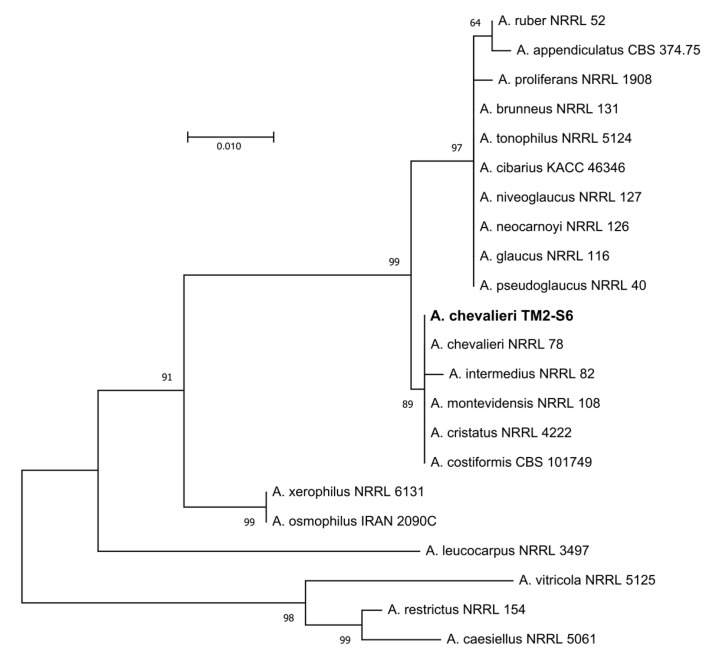
Maximum likelihood tree obtained from ITS rDNA sequence alignment of the isolate TM2-S6 and *Aspergillus* spp. of the section *Aspergillus restricti*. Reliability for internal branch is indicated when geater than 60%. *A. caesiellus*, *A. restrictus* and *A. crustosus* from section *Restricti* were used as outgroup. Genbank accessions are listed in supplementary Table S1. Scale represents substitutions per site.

According to the phylogenetic analyses of ITS rDNA sequence, the isolate TM2-S6 described in this study belongs to the *Aspergillus Restricti* section of the *Aspergillus* genus. Based upon blast search against the “rRNA_typestrains/ITS_RefSeq_Fungi” database and subsequent phylogeny analyses, TM2-S6 isolate was assigned as *Aspergillus chevalieri* with the GenBank number MT256106 (Figure 2).

**Figure 3:**
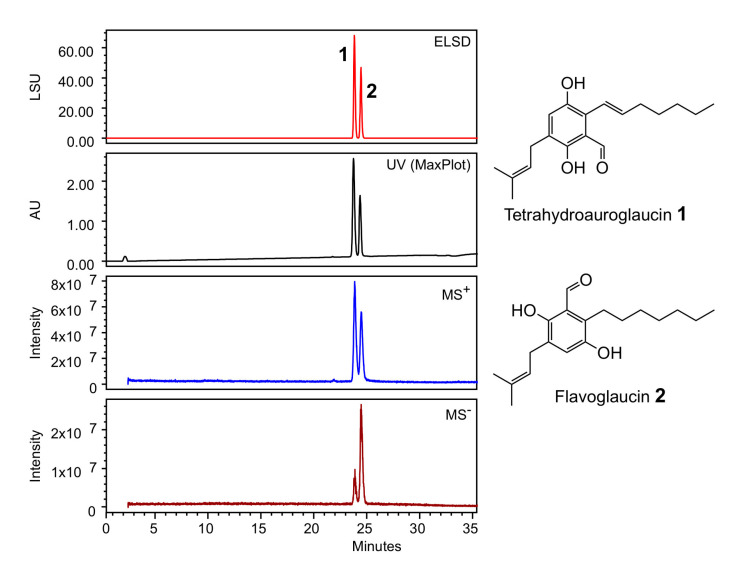
HPLC analysis of the ethyl acetate extract of *A. chevalieri* TM2-S6 cultivation broth. ELSD: Evaporative Light-Scattering Detector; UV Max-Plot: UV absorbance using a PhotoDiode Array detector, MS^+^, MS^−^: Mass Spectrometry detection in positive and negative mode, LSU: Light-Scattering arbitrary Unit, AU: arbitrary UV unit.

The strain was cultivated in PDB medium (Potato Dextrose Broth) composed of ingredients of plant origin. This medium is classified non-hazardous and is compatible with cosmetic formulations. After 9 days of culture, the medium was filtered, sterilized and lyophilized. The obtained powder was directly diluted to the desired concentration in the bioassay buffers, leading to the ACCB solution. Sample of ACCB solution was extracted with ethyl acetate and analyzed by HPLC coupled to a PhotoDiode Array detector (PDA), an evaporative light-scattering detector (ELSD) and a mass detector (MS). Figure 3 shows the presence of two peaks at 23,8 and 24,6 min. The peaks were dereplicated according to their ^1^H-NMR and HRMS spectra, and submitted to Antibase database of microbial compounds (Wiley-VCH) and natural compounds Reaxys database (Elsevier). Compound **1** was identified as tetrahydroauroglaucin and compound **2** as flavoglaucin (Supplementary Table S2, Figures S1 and S2). Both compounds have previously been isolated from *Aspergillus* strains; some are used in the preparation of the traditional Katsuobushi in Japanese cuisine [15,16].

### 2.2. Bioassays on Primary Human Fibroblasts

#### 2.2.1. Cell Viability in Vitro

**Figure 4:**
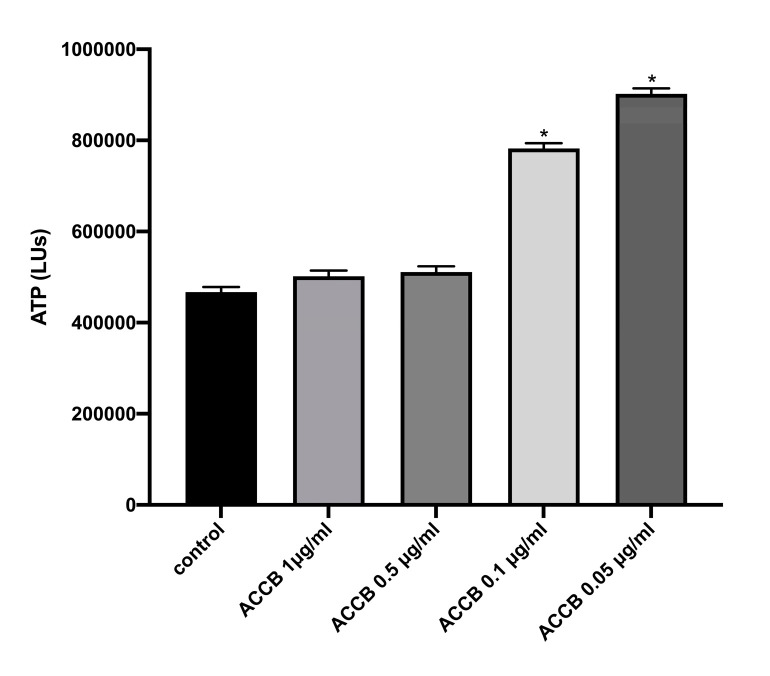
ATP levels (LUs) expressed as mean ± SEM for: control (untreated NHDF cells) and NHDF cells treated with four concentrations of ACCB (1 μg/mL, 0.5 μg/mL, 0.1 μg/mL, 0.05 μg/mL) * *p* < 0.05 significantly different from the control (ANOVA test).

Cell viability was assessed by ATP assay. We observed that lower concentrations exhibited increased ATP levels compared to control (*p* < 0.05) (Figure 4), indicating that cell viability is increased in lower concentrations of ACCB.

**Figure 5:**
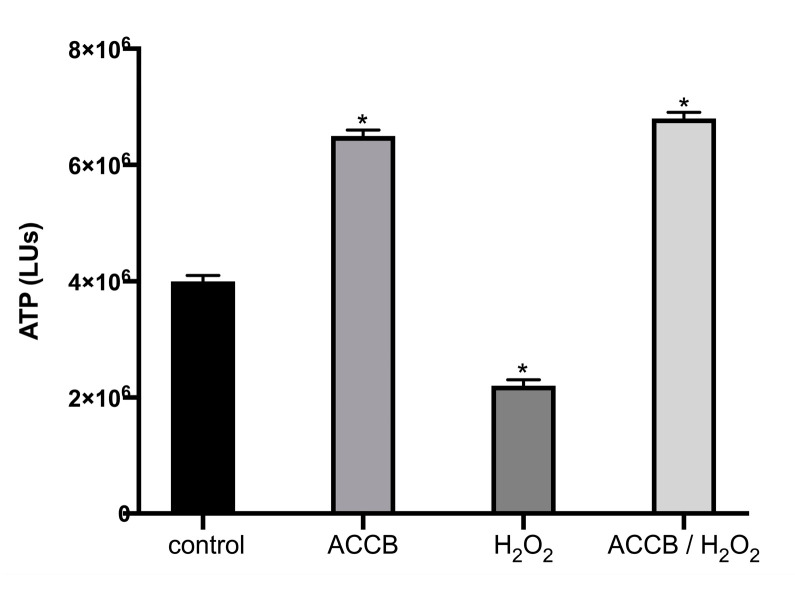
ATP levels (LUs) expressed as mean ± SEM for: control (untreated NHDF cells), ACCB (NHDF cells treated with 0.05 μg/mL ACCB, H_2_O_2_ (0.5 mM) and ACCB/H2O2 (NHDF cells treated first with 0.05 μg/mL ACCB and then with H_2_O_2_). * *p* < 0.05 significantly different from the control (ANOVA test).

Figure 5 shows the ATP levels of NHDF cells under or without oxidative stress. We observe that, compared to untreated cells, treatment with H_2_O_2_ decreased the cell viability (ATP levels) by 30%. However, the addition of ACCB (0.05 μg/mL) not only counteracts the toxicity induced by H_2_O_2_ but maintains the high level of cell viability as for the untreated cells.

#### 2.2.2. Genes Involved in Antioxidant Response Cell Pathway

**Figure 6:**
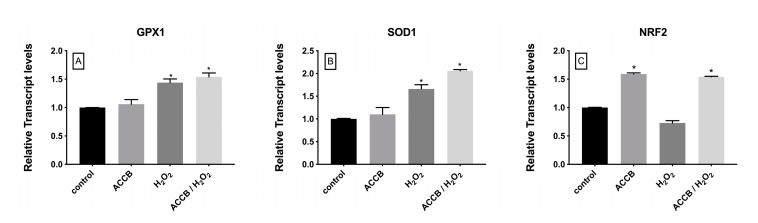
Relative expression of (**A**) GPX1, (**B**) SOD1, and (**C**) NRF2 in control (untreated NHDF), NHDF cells treated with ACCB (0.05 μg/mL) (ACCB), NHDF cells treated with H2O2 0.5 mM (H_2_O_2_), and NHDF cells treated with ACCB and H_2_O_2_ (0.5 mM) (ACCB/ H_2_O_2_). Transcript expression levels were obtained by qPCR and the means of ACTB and GADPH were used as internal references genes. The results are presented as a fold change ± SD respect to control and represent the mean ± SEM of three independent experiments. * *p* < 0.05 significantly different from control using one-way ANOVA.

To evaluate the antioxidant activity of ACCB we measured the expression of genes involved in the cellular antioxidant response. Figure 6A–B shows that the expression of glutathione peroxidase-1 (GPX1) and superoxide dismutase-1 (SOD1) increased under oxidative stress with or without addition of ACCB; in this case the contribution of ACCB remains limited. However, compared to control (*p* < 0.05), the expression of the nuclear factor, erythroid 2 like 2 (NRF2) was significantly increased in cells treated with ACCB with or without H_2_O_2_-induced oxidative stress (Figure 6C).

#### 2.2.3. Genes Involved in Cell Proliferation and Extracellular Matrix Organization

**Figure 7:**
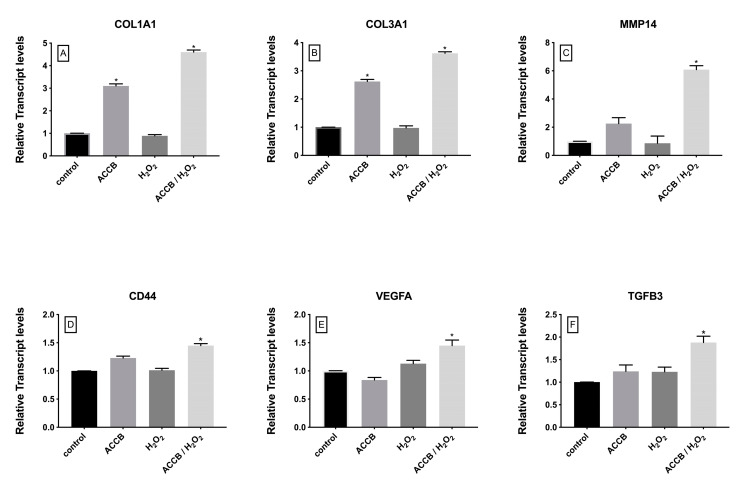
Relative expression of (**A**) COL1A1, (**B**) COL3A1, (**C**) MMP14, (**D**) CD44, (**E**) VEGFA, and (**F**) TGFB3 in control (untreated NHDF), NHDF cells treated with ACCB (0.05 μg/mL) (ACCB), NHDF cells treated with H2O2 0.5 mM (H_2_O_2_), and NHDF cells treated with ACCB and H_2_O_2_ (0.5 mM) (ACCB/H_2_O_2_). Transcript expression levels were obtained by qPCR and the mean of ACTB and GADPH were used as internal references genes. The results are presented as a fold change ± SD respect to control and represent the mean ± SEM of three independent experiments. * *p* < 0.05 significantly different from control using one- way ANOVA.

The impact of ACCB on the expression of genes involved in cell proliferation and extracellular matrix organization was also investigated. As shown in Figure 7, the expression of these genes in not impacted by the treatments of cells with H_2_O_2_, but significantly increased in ACCB treated cells, in the control and even more markedly in the H_2_O_2_ treated cells.

In detail, the expressions of collagen type I alpha 1 chain (COL1A1) and collagen type III alpha 1 chain (COL3A1) increased with the addition of ACCB under or without oxidative stress (Figure 7A,B). This significant increase is observed without oxidative stress, but is remarkably higher under H_2_O_2_ treatment. On the other hand, expressions of matrix metallopeptidase 14 (MMP14), CD44 molecule (CD44), vascular endothelial growth factor A (VEGFa) and transforming growth factor beta 3 (TGFB3) increased in ACCB treated NHDF cells only under oxidative stress compared to control (*p* < 0.05) (Figure 7C–F).

#### 2.2.4. Genes Involved in Cell Aging Pathway

**Figure 8:**
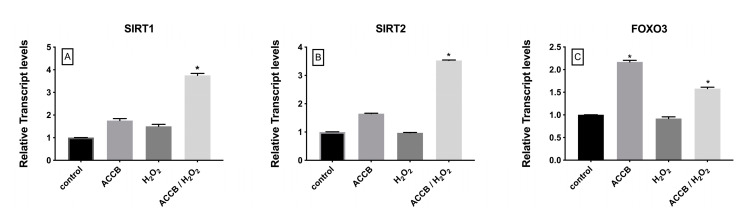
Relative expression of (**A**) SIRT1, (**B**) SIRT2, and (**C**) FOXO3 in control (untreated NHDF), NHDF cells treated with ACCB (0.05 μg/mL) (ACCB), NHDF cells treated with H2O2 0.5 mM (H_2_O_2_), and NHDF cells treated with ACCB and H_2_O_2_ (0.5 mM) (ACCB/ H_2_O_2_). Transcripts expression levels were obtained by qPCR and the mean of ACTB and GADPH were used as internal references genes. The results are presented as a fold change ± SD respect to control and represent the mean ± SEM of three independent experiments. * *p* < 0.05 significantly different from control using one-way ANOVA.

The expression of sirtuin 1 (SIRT1) and sirtuin 2 (SIRT2) increased significantly in ACCB treated H_2_O_2_-induced NHDF cells compared to control (*p* < 0.05) (Figure 8A,B) while expression of forkhead box O3 (FOXO3) increased in ACCB treated NHDF with or without oxidative stress (*p* < 0.05) (Figure 8C).

#### 2.2.5. Genes Involved in Inflammation

**Figure 9:**
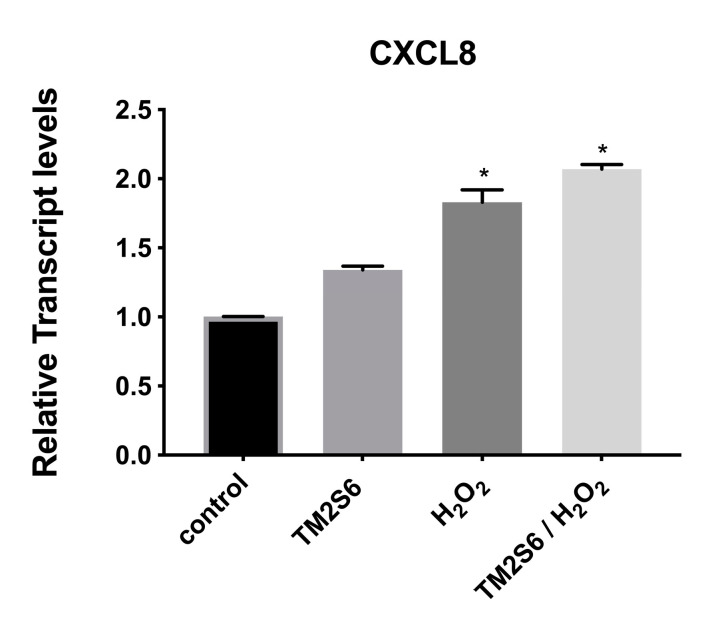
Relative expression of CXCL8 in control (untreated NHDF), NHDF cells treated with ACCB (0.05 μg/mL) (ACCB), NHDF cells treated with H2O2 0.5 mM (H_2_O_2_), and NHDF cells treated with ACCB and H_2_O_2_ (0.5 mM) (ACCB/H_2_O_2_). Transcript expression levels were obtained by qPCR and the mean of ACTB and GADPH were used as internal references genes. The results are presented as a fold change ± SD respect to control and represent the mean ± SEM of three independent experiments. * *p* < 0.05 significantly different from control using one-way ANOVA.

The expression of C-X-C motif chemokine ligand 8 (CXCL8) was measured, showing a limited increase in NHDF cells under oxidative stress with or without addition of ACCB (*p* < 0.05) (Figure 9).

## 3. Discussion

This is the first report on the biological cosmetic potential of *Aspergillus chevalieri* TM2-S6 cultivation broth extract (ACCB). This ingredient was prepared in media and with procedures compatible with cosmetic constraints (water as solvent, medium without animal constituents, no post-cultivation processing, only sterilizing filtration). In this study, we focused on the impact of ACCB on oxidative stress, simulated by H_2_O_2_ application on human fibroblast, which plays a key role in skin health and is sensitive to the deleterious effect of oxidative stress.

Before implementing molecular bioassays, we evaluated the impact of ACCB on cell viability by measuring the intracellular levels of ATP in NHDF cells. This experiment revealed that, at low concentrations of ACCB (0.05 µg/mL), ATP level is significantly enhanced, while even at high concentrations (1 µg/mL), no toxicity is observed compared to the control. This increase is associated with the enhancement of cell proliferation and energy metabolism of NHDF cells. Previous reports supported the conclusion that increased intercellular levels of ATP could be associated with higher levels of mitochondrial activity, energy metabolism as well as cell proliferation [17,18].

To gain insight on the molecular targets of ACCB, we investigated the expression of genes involved in skin biological pathways. According to the results reported in this paper, ACCB transcriptionally modulates genes involved in antioxidant, aging, extracellular matrix organization and less on inflammation.

In terms of antioxidant response, we investigated the expression of GXP1, SOD1 and NRF2 since these genes are considered key antioxidant markers [19,20,21,22]. It has been reported that GPX1, SOD1, NRF2 are up-regulated under oxidative stress [20,23,24,25]. Similarly, our results showed a significant yet limited increase in the expression of GPX1 and SOD1 under oxidative stress. Interestingly, the expression of NRF2 was significantly increased with the addition of ACCB compared to control, regardless of the addition of H_2_O_2_. This outcome suggests a multifactorial antioxidant protective role of ACCB. The activity of ACCB on NFR2 without external oxidant damage by H_2_O_2_ could be considered as a preventive or a pro-survival action mediated by the induction of a global detoxification pathway [26].

In terms of aging pathway, we investigated the expression levels of SIRT1 and SIRT2 since sirtuins are mediators in cellular aging process either by delaying the cellular senescence or by expanding cells lifespan through the regulation of different cellular functions [27]. Our results demonstrated a significant upregulation in the expression of SIRT1 and SIRT2 in NHDF cells treated with ACCB under oxidative stress as compared with control. This upregulation is in agreement with previous reports [28,29]. In addition, we investigated the expression of FOXO3 as it has a significant regulatory role in cellular aging process as well as antioxidant response [30,31,32,33]. FOXO3 was upregulated upon treatment with ACCB with or without H_2_O_2_ treatment of NHDF cells. As for NFR2, this effect seems independent from exogenous oxidative stress with H_2_O_2_ and may be related to the preventive and protective role already reported for FOXO3 [34].

Extracellular matrix (ECM) organization has an important role in many cellular processes. In this study, we focused on the in vitro expression of COL1A1, COL3A1, MMP14 responsible for the main modulations in ECM during cellular aging process [35]. Specifically, COL1A1 and COL1A2, together, encode the type I collagen, an abundant extracellular protein in the skin [36], while COL3A1 encodes the type III collagen, an important protein for skin balance, together with collagen type I [37]. In addition, MMP14 is responsible for collagen degradation, and it has been reported that MMP14 expression is upregulated under oxidative stress [24]. Our results indicated a significant upregulation in the expression of COL1A1 and COL3A1 with the addition of ACCB with or without oxidative stress. This behavior potentially underlines that ACCB protects collagen conformation during cellular aging process. The increased expression of MMP14, in accordance with a previous report [38], corroborates the crucial role of ACCB in skin aging biological mechanism. Furthermore, we also investigated the expression of the gene CD44 as it regulates pro-proliferative as well as migratory effects of cells in high hyaluronic-abundant tissues [39]. Our results showed a significant upregulation of CD44 expression in NHDF cells treated with ACCB under oxidative stress. This potentially indicates that ACCB stimulates the production of hyaluronic acid by fibroblasts under oxidative stress.

The expression of VEGFα and TGFB3 was modulated significantly under our experimental conditions. VEGFα plays a key role in the regulation of cell proliferation during the angiogenesis process [40,41] and TGFB3 is involved in cell migration [42]. Our results showed significant increased expression of VEGFα and TGFB3 in fibroblasts treated with ACCB under oxidative stress. These results are in accordance with previous studies [43,44].

Finally, the impact of ACCB on inflammation is very limited as among other genes investigated, only the expression level of CXCL8 gene was slightly increased. CXCL8 belongs to the cytokine family with a significant role in inflammation [45].

*Aspergillus chevalieri* was reported in literature to produce flavoglaucin, a strong antioxidant and radical scavenger compound [15,46]. ACCB ingredient analysis by HPLC coupled to PDA, ELSD and MS detectors highlighted the presence of two main compounds, tetrahydroauroglaucin and flavoglaucin who may account for the bioactivities reported in this paper [15,16].

Future investigations are focused on the precise contribution of each of these compounds in the antioxidant role of ACCB.

## 4. Materials and Methods

### 4.1. Strain Isolation and Identification

*Aspergillus chevalieri* TM2-S6 was isolated from a 1 cm^3^ sample of the sponge *Axinella* collected on the upper mesophotic zone off Tel Aviv–Jaffa, Israeli Mediterranean coast (32°1’42.431″ N 34°46’42.323″ E, 38 m depth). The sponge was collected at 38 m depth by a ROV (Remote Operating Vehicule) equipped with a collection arm (the sample is part of the TASCMAR project collection, EU H2020, grant agreement 634674). The collection permit #2016/41271 was delivered by the “Israel Nature and National Parks Protection Authority”. The sponge sample was frozen on-boat and stored until further processing. The sample was ground in sterile sea-water and heated at 50 °C for 1 h. The suspension was serially diluted, plated on selective isolation media, and incubated at 28 °C for at least 6 weeks. The strain was isolated from marine agar medium. The colony was purified on potato dextrose broth agar (PDBag, Difco, Fisher Scientific, F67403 Illkirch CEDEX, France) and marine broth agar (MBag, Difco) media and preserved in 10% glycerol solution.

### 4.2. Phylogeny Investigation

Genomic DNA isolation and amplification of the ITS region was performed as described previously [13]. Phylogeny inference was performed using MEGA X [47] and comprised the following steps. Sequences from TM2-S6 and *Aspergillus* spp. of the section *Aspergillus* and *Restricti* previously described [48] and aligned with MUSCLE [49]. After alignment, ambiguous regions were removed with Gblocks (v0.91b) [50]. The evolutionary history was inferred using the maximum likelihood method and Kimura 2-parameter model [51]. Reliability for internal branch was assessed using the bootstrap method with 1000 replicates. The tree with the highest log likelihood is shown. Accordingly, the strain was named *Aspergillus chevalieri* TM2-S6.

### 4.3. Cultivation and Extract Preparation

*Aspergillus chevalieri* TM2-S6 spores were conserved at −20 °C in 10% glycerol. Before cultivation, the strain was revived for 5 days on a 15 cm petri plate containing potato dextrose agar (PDBag). Sterile water (4 × 10 mL) was poured on the plate surface, and the spores were recovered from the plates by gentle scratching of the surface with a scalpel. Three plates offer 100 mL of concentrated spore suspension. This inoculum was used to cultivate the strain *A. chevalieri TM2-S6* in a 20 × 2 L Erlenmeyer flask containing 1 L of potato dextrose broth medium (PDB, Difco, Fisher Scientific, F67403 Illkirch CEDEX, France over 9 days in a rotary shaker at 28 °C and 130 rpm. The culture broth was filtrated and sterilized using 0.22 µm sterile filtration units. Two liters of the filtrated culture broth were then lyophilized to give 8.25 g of crude *A. chevalieri TM2-S6* cultivation broth extract (ACCB). ACCB is freely soluble in aqueous media, which is suitable for cosmetic investigations. ACCB was solubilized in NHDF cultivation media.

### 4.4. Human Skin Cell Culture

Primary Normal Human Dermal Fibroblasts (NHDF) were purchased from Lonza Clonetics TM (Lonza, Walkersville, MD, USA) [52]. NHDF cells were cultured according to Lonza instructions in FGM™ 2 BulletKit™ mediun (Lonza, Walkersville, MD, USA) supplemented with 2% serum.

### 4.5. Cell Viability Assay

NHDF cells were incubated for 48 h with four concentrations of ACCB (1 μg/mL, 0.5 μg/mL, 0.1 μg/mL, 0.05 μg/mL) and then intracellular levels of ATP were measured with ViaLight HS BioAssay kit (Lonza). The same procedure was followed for the determination of ATP levels in NHDF cells treated with 0.05 μg/mL under oxidative stress. The experiments were performed in a single-tude luminator (GloMax 20/20, Promega). Three independent experiments were performed (8 technical repetitions).

### 4.6. H_2_O_2_ Treatment

The treatment of NHDF cells with *H_2_O_2_* was performed according to a protocol described previously [13]. Moreover, the doses of H_2_O_2_ and incubation time was selected according to previously reported works [53,54].

### 4.7. Gene Expression Analysis by Real-Time RT-qPCR 

#### 4.7.1. RNA Extraction and cDNA Synthesis

For total RNA (tRNA) extraction, the Nucleospin RNA kit (Macherey-Nagel) was used following the manufacturer’s instructions. For the experiments, 500 ng of tRNA was used. For the synthesis of complimentary DNA (cDNA), the PrimeScript-RT reagent kit (Takara) was used.

#### 4.7.2. RT-qPCR Analysis

qPCR was used to analyze the mRNA expression of genes modulated by ACCB under our experimental conditions as described before [13,53]. The qPCR reactions were performed in three independent biological repetitions (six technical replicates). Our results were based on the experimental conditions: untreated NHDF cells (control), NHDF cells treated with ACCB (0.05 μg/mL), NHDF cells treated with 0.5 mM H_2_O_2_ (H_2_O_2_), NHDF cells treated with ACCB (0.05 μg/mL) and 0.5 mM H_2_O_2_ (ACCB/H_2_O_2_) (Supplementary Tables S3 and S4).

### 4.8. Statistical Analysis

One-way analysis of variance (ANOVA) was used to assess the interactions among the gene expression data on different experimental states. Multiple comparison was used for type I error likelihood. Normal distribution was confirmed for all data. All the statistical analysis was performed on SPSS 17.0 (SPSS Inc., Chicago, IL, USA) with a statistical significance level of 5%.

## Supplementary Materials

The following are available online at https://www.mdpi.com/1660-3397/18/9/460/s1, Table S1: *Aspergillus species of the section Aspergillus restricti witn Genbank numbers*. Table S2. Materials and methods used for analytical and structural characterization. Table S3. Relative mRNA expression ration versus control into three different treatments. Table S4. Characteristics and references of the investigated genes. Figure S1. ^1^H NMR and HRMS Spectra of tetrahydroauroglaucin. Figure S2. ^1^H NMR and HRMS Spectra of flavoglaucin.
