# Supplementary material for: Marine Fungus Aspergillus chevalieri TM2-S6 Extract Protects Skin Fibroblasts from Oxidative Stress
Source: Mar Drugs. 2020 Sep 8;18(9):460. doi: 10.3390/md18090460 (PMC7551392; doi:10.3390/md18090460)
Supplement: Supplementary file 1 [file marinedrugs-18-00460-s001.pdf]

Marine fungus *Aspergillus chevalieri* TM2-S6 Extract protects skin fibroblasts from oxidative stress

Sophia Letsiou[1*], Artemis Bakea[1], Géraldine Le Goff[2], Philippe Lopes[2], Michal Weis[3], Yehuda Banayahu[3], Jamal Ouazzani[2].

1. Laboratory of Biochemistry, Research and Development department, APIVITA S.A., Industrial Park of Markopoulo Mesogaias, 19003 Markopoulo Attiki, Athens, Greece.
2. Institut de Chimie des Substances Naturelles ICSN, Centre National de la Recherche Scientifique CNRS, Avenue de la Terrasse 91198, Gif-sur-Yvette, France.
3. School of Zoology, George S. Wise Faculty of Life Sciences, Tel Aviv University, Ramat Aviv, Tel Aviv 69978, Israel.

Supplementary Material

Species	Section	Genbank (Samson et al. 2014)	strain
A. pseudoglaucus	Aspergillus	EF652050	NRRL 40
A. neocarnoyi	*Aspergillus*	EF652057	NRRL 126
A. cibarius	*Aspergillus*	JQ918177	KACC 46346
A. brunneus	*Aspergillus*	EF652060	NRRL 131
A. glaucus	*Aspergillus*	EF652052	NRRL 116
A. tonophilus	*Aspergillus*	EF652081	NRRL 5124
A. niveoglaucus	*Aspergillus*	EF652058	NRRL 127
A. proliferans	*Aspergillus*	EF652064	NRRL 1908
A. appendiculatus	*Aspergillus*	HE615132	CBS 374.75
A. ruber	*Aspergillus*	EF652066	NRRL 52
A. montevidensis	*Aspergillus*	EF652077	NRRL 108
A. cristatus	*Aspergillus*	EF652078	NRRL 4222
A. chevalieri	*Aspergillus*	EF652068	NRRL 78
A. costiformis	*Aspergillus*	HE615136	CBS 101749
A. intermedius	*Aspergillus*	EF652074	NRRL 82
A. xerophilus	*Aspergillus*	EF652085	NRRL 6131
A. osmophilus	*Aspergillus*	KC473921	IRAN 2090C
A. leucocarpus	*Aspergillus*	EF652087	NRRL 3497
A. restrictus	*Restricti*	EF652042	NRRL 154
A. caesiellus	*Restricti*	EF652044	NRRL 5061
A. vitricola	*Restricti*	EF652046	NRRL 5125
A. chevalieri	***Restricti***	**MT256106** (Genbank, this paper)	**TM2-S6**

Table S1. *Aspergillus* species of the section *Aspergillus restricti* witn Genbank numbers

Analytical HPLC system consisted of an Alliance Waters 2695 co ntroller coupled with a PhotoDiode Array Waters 2996, an evaporative light-scattering detector ELSD Waters 2424 detector and a mass detector Waters QDa (Waters SAS, Saint-Quentin-en-Yvelines, France). Sunfire C_{18} column (4.6 × 150 mm, 3.5 μm) was used with a flow rate of 0.7 mL/min. The elution gradient consisted of a linear gradient from 100 % solvent A to 100% solvent B in 40 min, then 10 min at 100% B (Solvent A: H_2O + 0,1 HCOOH, Solvent B: ACN + 0,1% HCOOH).
Preparative HPLC was performed on a semi-preparative Sunfire C_{18} column (10 × 250 mm, 5 μm) using a Waters autosampler 717, a pump 600, a photodiode array detector 2996 and an ELSD detector 2420 ((Waters SAS, Saint-Quentin-en-Yvelines, France). ACCB medium of *Aspergillus chevalieri TM2-S6* was extracted with ethyl acetate leading to the mixture of two main compounds reported in the results section.
NMR experiments were performed using a Bruker Avance III 600 MHz spectrometer equipped with a TCi cryo-probe head for compounds **8**, **9** and **10**, and a Bruker Avance 500 MHz spectrometer for compounds **1 to 7** (Bruker, Vienna, Austria). The spectra were acquired in CD_3OD (δ_H 3.31 ppm and δ_C 49.15 ppm), in CD_2Cl_2 (δ_H 5.32 ppm and δ_C 53.10 ppm) or in Acetone-d_6 (δ_H 2.04 ppm and δ_C 29.8 ppm and 206.5 ppm) at 300K.
High-resolution mass spectra were obtained on a Waters LCT Premier XE spectrometer equipped with an ESI-TOF (electrospray-time of flight) by direct infusion of the purified compounds (Waters SAS, Saint-Quentin-en-Yvelines, France).

Table S2. Materials and methods used for analytical and structural characterization

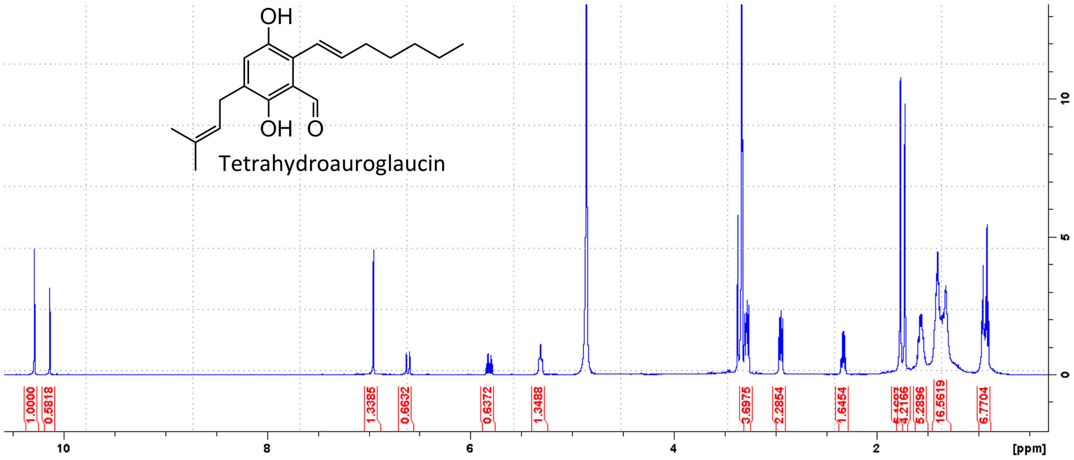

CD$_3$OD, 500 MHz

Elemental Composition Report

Single Mass Analysis
Tolerance = 10.0 PPM / DBE: min = -1.5, max = 100.0
Element prediction: Off
Number of isotope peaks used for i-FIT = 9

Monoisotopic Mass, Even Electron Ions
467 formula(e) evaluated with 3 results within limits (all results (up to 1000) for each mass)
Elements Used:
C: 0-50 H: 0-100 N: 0-10 O: 0-20
29-Aug-2019 11:35:27
2: TOF MS ES+

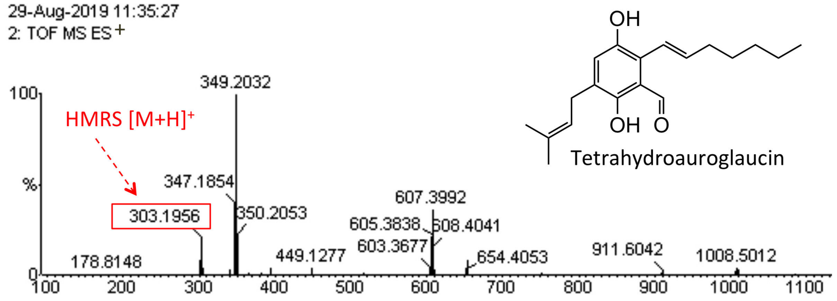

Minimum: -1.5
Maximum: 5.0 10.0 100.0

Mass	Calc. Mass	mDa	PPM	DBE	i-FIT	i-FIT (Norm)	Formula
303.1946	303.1933	1.3	4.3	7.5	50.9	0.4	C15 H23 N6 O
	303.1960	-1.4	-4.6	6.5	52.6	2.1	C19 H27 O3
	303.1920	2.6	8.6	2.5	52.0	1.5	C14 H27 N2 O5

Figure S1. ^{1}H NMR and HRMS Spectra of tetrahydroauroglaucin

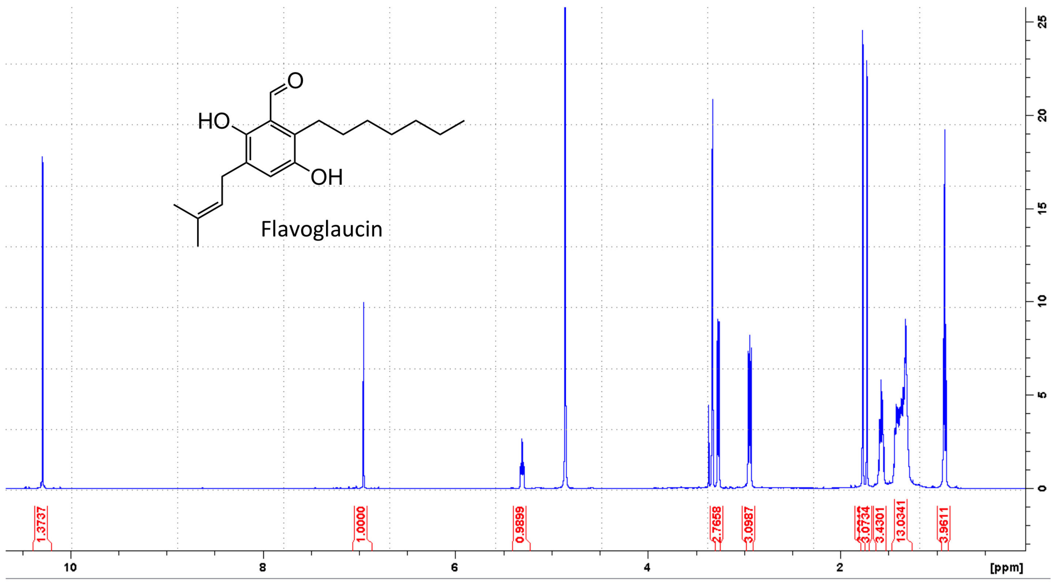

CD_3OD, 500 MHz

1H NMR Spectra of **2**

Elemental Composition Report

Single Mass Analysis

Tolerance = 10.0 PPM / DBE: min = -1.5, max = 100.0
Element prediction: Off
Number of isotope peaks used for i-FIT = 9

Monoisotopic Mass, Even Electron Ions
472 formula(e) evaluated with 3 results within limits (all results (up to 1000) for each mass)
Elements Used:
C: 0-50 H: 0-100 N: 0-10 O: 0-20
29-Aug-2019 11:38:26
1: TOF MS ES

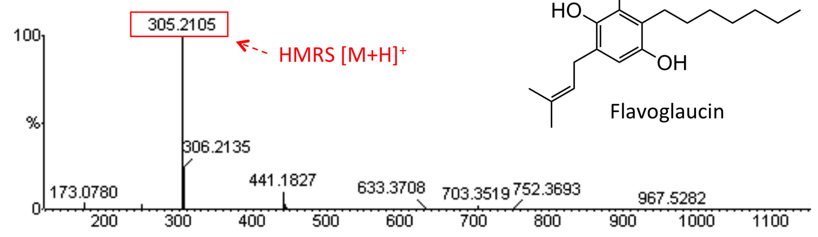

Minimum:						-1.5	
Maximum:			5.0		10.0	100.0	
Mass	Calc. Mass	mDa	PPM	DBE	i-FIT	i-FIT (Norm)	Formula
305.2105	305.2117	-1.2	-3.9	5.5	449.5	0.0	C19 H29 O3
	305.2090	1.5	4.9	6.5	456.0	6.6	C15 H25 N6 O
	305.2076	2.9	9.5	1.5	457.7	8.2	C14 H29 N2 O5

Figure S2. ^{1}H NMR and HRMS Spectra of flavoglaucin

Gene	Gene ID	Relative mRNA expression ration versus control			P value
		NHDF treated with ACCB 0.05 μg/ml	NHDF treated with H_2O_2	NHDF treated with H_2O_2 and ACCB 0.05 μg/ml	
CXCL8	3576	1.34	1.83	2.07	0.03
CD44	960	1.23	1.01	1.49	0.04
COL1A1	1277	3.13	0.89	4.60	0.02
COL3A1	1281	2.62	0.97	3.68	0.02
FOXO3	2309	2.17	0.92	1.58	0.04
GPX1	2876	1.16	1.49	1.54	0.04
MMP14	4323	2.26	0.84	6.08	0.01
NRF2	4780	1.59	0.74	1.54	0.04
SIRT1	23411	1.32	1.20	3.75	0.02
SIRT2	22933	1.43	0.97	3.53	0.02
SOD1	6646	1.10	1.66	2.16	0.03
VEGFA	7422	0.74	1.23	1.50	0.04
TGFB3	7043	1.24	1.23	1.88	0.04

Table S3. Relative mRNA expression ration versus control into three different treatments

Gene Symbol	Gene Name	Accession No	KEGG pathway	Primmer F (5'-3')	Primmer R (5'-3')
ACTB	actin. beta	NM_001101.3	hsa04810	CTGTCCACCTTC CAGCAGATGT	AGCATTTGCGG TGGACGAT
GAPDH	glyceraldehyde-3-phosphate dehydrogenase	NM_001256799.2	hsa01200	TTGCCCTCAACG ACCACTTT	CACCCTGTTGCT GTAGCCAAA
CD44	CD44 molecule	NM_000610.3	hsa04512	CCAGAAGGAAC AGTGGTTTGGC	ACTGTCCTCTG GGCTTGGTGTT
COL1A1	collagen. type I. alpha 1	NM_000088.3	hsa04512	CCTCAAGATGTG CCACTCTGA	CTTTGATGGCAT CCAGGTTG
COL3A1	collagen. type III alpha 1	NM_001303110.1	hsa04512	GGAGTTTAGAAG TGCGCCATTC	CCAAAAGCTGT AAGCGTTTGC
FOXO3	forkhead box O3	NM_001455.3	hsa04068	CAGGCACCATG AATCTGAATGA	GGAGCGTGATG TTATCCAGCA
TGFB3	transforming growth factor beta 3	NM_001329938.2	hsa04068	CTAAGCGGAATG AGCAGAGGATC	TCTCAACAGCC ACTCACGCACA
GPX1	glutathione peroxidase 1	NM_000581.2	hsa00480	CGATGTTGCCTG GAACTTTGAG	ATGTCAATGGT CTGGAAGCGG
CXC8	chemokine (C-X-C motif) ligand 8	NM_000584.3	hsa04064	ACACTGCGCCAA CACAGAAAT	ACTTCTCCACA ACCCTCTGCAC
MMP14	matrix metallopeptidase 14	NM_004994.2	hsa04668	ACGACGTCTTCC AGTACCGAGA	TAGGTCACGTA GCCCACTTGGT
NRF2	nuclear factor. erythroid 2-like 2	NM_001145412.3	hsa04141	CCAAAGAGCAG TTCAATGAAGC	GCAGCCACTTT ATTCTTACCCC
SIRT1	sirtuin 1	NM_001142498.1	hsa04068	GGAGCAGATTA GTAGGCGGCTT	GCGCCATGGAA AATGTAACG
SIRT2	sirtuin 2	NM_001193286.1	hsa007608	TGACGCCCAAGT GTGAAGACT	ATACAGGAGAA GAAACGCGCTG
SOD1	superoxide dismutase 1	NM_000454.4	hsa04146	GGATGAAGAGA GGCATGTTGGA	TAGACACATCG GCCACACCAT
VEGFA	vascular endothelial growth factor A	NM_001025366.2	hsa04370	CAGACGTGTAAA TGTTCCTGCA	ACGTTCGTTTA ACTCAAGCTGC

Table S4. Characteristics and references of the investigated genes
